# Attentional Biases and Nonsuicidal Self-Injury Urges in Adolescents

**DOI:** 10.1001/jamanetworkopen.2024.22892

**Published:** 2024-07-18

**Authors:** Andreas Goreis, Bettina Pfeffer, Carola Hajek Gross, Diana Klinger, Sofia M. Oehlke, Heidi Zesch, Laurence Claes, Paul L. Plener, Oswald D. Kothgassner

**Affiliations:** 1 Department of Child and Adolescent Psychiatry, Medical University of Vienna, Vienna, Austria; 2 Comprehensive Center for Pediatrics (CCP), Medical University of Vienna, Vienna, Austria; 3 Clinical Psychology, Faculty of Psychology and Educational Sciences, KU Leuven, Leuven, Belgium; 4 Faculty of Medicine and Health Sciences, University of Antwerp, Antwerp, Belgium; 5 Department of Child and Adolescent Psychiatry and Psychotherapy, University of Ulm, Ulm, Germany

## Abstract

**Importance:**

Nonsuicidal self-injury (NSSI) is a significant clinical concern among adolescents. Exposure to NSSI-related content on social media platforms has been suspected to potentially act as a trigger for NSSI.

**Objective:**

To use free-viewing eye-tracking and dot-probe paradigms to examine attentional bias and psychophysiological responses to NSSI-related pictorial and textual stimuli in adolescents with and without a history of NSSI.

**Design, Setting, and Participants:**

From June 2022 to April 2023, adolescent participants in Vienna, Austria with and without a history of NSSI were exposed to NSSI-related stimuli in this nonrandomized controlled trial. Data were analyzed from December 2023 to January 2024.

**Exposure:**

Exposure to NSSI-related stimuli.

**Main Outcomes and Measures:**

During both tasks, subjective arousal, NSSI urges, and autonomic nervous system activity were assessed.

**Results:**

A total of 50 adolescents in 2 groups, 25 who engaged in NSSI (mean [SD] age 15.86 [1.14] years; 19 female participants [76%]) and 25 who did not (mean [SD] age 16.40 [1.71] years; 19 female participants [76%]) were included. Adolescents with a history of NSSI—but not those without a history of NSSI—showed a clear attentional bias toward NSSI-related images during eye-tracking, as indicated by increased initial fixations (500 ms stimulus presentation mean difference, 28.64%; 95% CI, 18.31%-38.98%; *P* < .001; 1000 ms stimulus presentation mean difference, 18.50%; 95% CI, 9.05%-27.95%; *P* < .001) and longer fixation durations (500 ms mean difference, 29.51 ms; 95% CI, 4.3-54.72 ms; *P* < .001; 1000 ms mean difference, 39.83 ms; 95% CI, 6.90-72.76 ms; *P* < .001), regardless of stimulus duration. This bias was associated with a heightened urge to engage in NSSI (*d* = 1.22; 95% CI, 0.69-1.73; *P* < .001), a trend not seen in adolescents without a history of NSSI. Similarly, in the dot-probe task, only the NSSI group showed an attentional bias toward NSSI images but not toward trauma images, emphasizing the specificity of their attentional bias. Physiological measures revealed no significant differences, suggesting that viewing NSSI images is not associated with increased autonomic arousal. Textual NSSI content did not provoke an attentional bias or heighten NSSI urges in either group.

**Conclusions and Relevance:**

In this nonrandomized controlled trial of 50 adolescents, results highlighted a specific attentional bias toward NSSI-related pictorial stimuli in adolescents with a history of NSSI, particularly a difficulty in disengaging from NSSI images. These findings contribute to understanding maladaptive information processing in NSSI and suggest implications for clinical management and cognitive models addressing NSSI triggers.

**Trial Registration:**

German Clinical Trials Register identifier: DRKS00025905.

## Key Points

**Question:**

Do pictorial and textual stimuli with nonsuicidal self-injury (NSSI) content trigger urges to engage in NSSI or physiological stress in adolescents who engage in NSSI?

**Findings:**

In this nonrandomized controlled trial of 50 adolescents, those with a history of NSSI exhibited an attentional bias toward NSSI-related images, which was associated with increased urges to engage in NSSI, unlike adolescents without a history of NSSI. No significant autonomic arousal or attentional bias toward textual NSSI content was observed in either group.

**Meaning:**

These findings suggest a specific attentional bias toward NSSI imagery in adolescents with a history of NSSI that is associated with increased urges, a bias not observed in adolescents without a history of NSSI.

## Teaser

This nonrandomized controlled trial evaluates the use of free-viewing eye-tracking and dot-probe paradigms to examine attentional bias and psychophysiological responses to nonsuicidal self-injury–related stimuli in adolescents with and without a history of nonsuicidal self-injury.

## Introduction

Nonsuicidal self-injury (NSSI) is the deliberate, repetitive infliction of bodily harm without suicidal intent.^1^ NSSI frequently co-occurs with various mental disorders, such as depressive disorder, posttraumatic stress disorder (PTSD), and borderline personality disorder.^2^ Recognized as a transdiagnostic entity, NSSI has been proposed as a potential disorder warranting additional research in the *Diagnostic and Statistical Manual of Mental Disorders* (Fifth Edition) (DSM-5).^3^ Global prevalence estimates of NSSI among adolescents are approximately 16 to 17%.^4,5^ NSSI behaviors include cutting, scratching, burning, and striking or punching objects to cause bruising or bleeding.^6^ Notably, NSSI is a significant predictor of subsequent suicide attempts.^7^ Among other functions, NSSI serves to regulate emotions or manage stress.^8,9^ While progression has been made in elucidating the biological^10,11^ and social^12^ predictors of NSSI, alongside functions of NSSI,^13,14^ research on its psychophysiological mechanisms of onset and triggers is lacking.

While pictures of NSSI are displayed on social media,^15^ concerns have been raised that exposure to NSSI-related stimuli (eg, depictions of wounds or scars or text concerning NSSI) may have detrimental effects and may even trigger NSSI behaviors.^16^ Evidence^17^ suggests that viewing NSSI content—especially images and videos—on social media can precipitate urges and actual acts of NSSI, although these studies often rely on retrospective self-reports. Individuals with a history of NSSI often report experiencing distressing emotions and unpleasant physiological responses upon viewing NSSI content online, potentially instigating or exacerbating NSSI behaviors.^18^ Confirming this notion, a study^19^ found that NSSI-related pictures were more arousing to female adolescents with a history of NSSI than to those without a history of NSSI.

Few studies have differentiated the type of content that triggers NSSI urges, focusing on images vs text. Most participants identified images, not text, as driving online NSSI content pursuit^18^ and triggering urges.^20^ Nevertheless, the qualitative findings of the latter study suggest that a minority of participants perceived textual representations as more vivid and, consequently, more likely to trigger urges. Comments accompanying NSSI images on social media platforms have also been suggested to act as a form of social contagion through reinforcement mechanisms.^15^

An additional line of research explores whether individuals who engage in NSSI allocate special attention to NSSI stimuli. In various psychopathologies, people tend to focus on disorder-related stimuli over neutral ones. For example, anxiety disorders are characterized by biases toward threat-related stimuli (eg, angry faces), and depressive disorders toward negative stimuli (eg, sad facial expressions).^21,22^ This attentional bias has also been observed in adolescent samples with mood disorders.^23^ Individuals with NSSI may similarly show a bias toward NSSI-related depictions, measurable using dot-probe tasks,^24^ which compare reaction times to probes replacing neutral or emotionally charged stimuli, suggesting attentional preference or avoidance. Two studies using a dot-probe paradigm showed that adults with NSSI exhibit an attentional bias toward negative^25^ and NSSI-related stimuli.^26^

Manual reaction time measures such as the Stroop task^27,28^ are useful for studying attentional biases, but eye-tracking technology provides additional insights into overt and covert components.^29^ Eye-tracking differentiates between automatic vigilance^30^ and more strategic avoidance. Automatic vigilance is defined as initial fixations on certain stimuli, while strategic disengagement is measured by fixation durations. The vigilance-avoidance model posits initial orientation toward threatening stimuli, followed by arousal and subsequent avoidance to mitigate arousal.^31^ Although preliminary evidence of attentional bias exists,^26^ a comprehensive investigation into autonomic arousal in those with NSSI, particularly regarding NSSI and threat words, is needed. Hypervigilance toward threat stimuli increases psychological responses like stress and tension, as well as physiological arousal. Since arousal and NSSI urges may be triggered by NSSI-related stimuli but not negatively valenced stimuli,^19,26^ it would be insightful to see if threat or trauma-related words also trigger these urges.

Accordingly, this study aimed to elucidate mechanisms of NSSI engagement and maintenance in adolescents with a history of NSSI using eye-tracking (with word and text stimuli) and a dot-probe task with NSSI-related, threat, and neutral stimuli. We assessed subjective arousal (perceived stress and tension), NSSI urges, and autonomic nervous system (ANS) activity (heart rate and skin conductance). Adolescents with a history NSSI were compared with a control group with no history of NSSI to investigate attentional bias to NSSI words and pictures. We hypothesized that (1) adolescents who engaged in NSSI would exhibit more first fixations, extended fixation duration times, and more skin conductance responses to NSSI words and pictures compared with neutral words and pictures in an eye-tracking free-viewing task relative to those with no history of NSSI; (2) adolescents who engaged in NSSI would demonstrate elevated levels of stress, tension, and NSSI urge, as well as higher skin conductance level and heart rate in response to exposure to NSSI stimuli in comparison with those with no history of NSSI; (3) adolescents who engaged in NSSI would exhibit pronounced attentional bias toward NSSI stimuli in the dot-probe task relative to those with no history of NSSI.

## Methods

This nonrandomized controlled trial was approved by the ethics committee of the Medical University of Vienna. Written informed consent was obtained from participants and their legal guardians before participation. The trial protocol is presented in Supplement 1. This study was conducted according to the Transparent Reporting of Evaluations With Nonrandomized Designs (TREND) reporting guideline.^32^

### Participants

Participants (aged 14-18 years) were recruited from various sources to form 2 groups: individuals who engaged in NSSI and individuals who did not. Participants with a history of NSSI or who were currently engaged in NSSI were predominantly inpatients and outpatients at the Department of Child and Adolescent Psychiatry, Medical University of Vienna, Austria. Participants with no history of NSSI were recruited from the general public and schools in Vienna and nearby areas. Recruitment spanned from July 2022 to May 2023. Details on recruitment strategy, inclusion criteria, participant flow, and power analysis are in eMethods 1 in Supplement 2, and the TREND flowchart is in eFigure 1 in Supplement 2.

### Procedure

The study was conducted at the Department of Child and Adolescent Psychiatry, Medical University of Vienna, Austria. After attaching heart rate and skin conductance devices, participants sat quietly for 5 minutes to obtain baseline levels (eFigure 2 in Supplement 2 for the study procedure). Participants received instructions and sat 60 cm from the eye-tracking monitor (Tobii TX Display, Tobii Instruments). The dot-probe task followed the free-viewing tasks. Afterward, clinical interviews and self-report questionnaires on NSSI, PTSD, stress, and depressive symptoms were completed. Momentary stress, tension, and NSSI urges were assessed using 5-point Likert scales (ranging from 1, not at all, to 5, extremely) at 9 time points (eFigure 2 in Supplement 2). Participants were then debriefed, thanked, and given a €25 voucher.

### Measures

Participant race and gender were assessed by verbally inquiring about their self-identification for the purposes of sample description only. We used the German Version of the revised Self-Injurious Thoughts and Behaviors Interview^33^ to assess NSSI presence, frequency, and characteristics. For PTSD symptoms, we used the German versions of the Child and Adolescent Trauma Screen 2^34^ and the Clinician-Administered PTSD Scale for *DSM-5* in Children and Adolescents,^35^ given the inclusion of PTSD-related stimuli in the dot-probe task. The German version^36^ of the Perceived Stress Scale^37^ indicated perceived stress over the last month, and the German version^38^ of the Beck Depression Inventory II^39^ evaluated depressive symptoms over the past 2 weeks (see eMethods 2 in Supplement 2 for detailed instrument descriptions).

### Free-Viewing Task

The free-viewing task consisted of 2 parts: in the first part, 4 words were presented on the eye-tracking monitor across 64 trials. Thirty-two trials featured 3 neutral words (eg, bread or coating) and 1 NSSI-related word (eg, cutting or blood), while the remaining 32 trials contained 4 neutral words. In the second part, the free-viewing picture task, 4 pictures were presented across 64 trials. Similarly, 32 trials featured 3 neutral pictures along with 1 NSSI-related picture, and the other 32 trials consisted of 4 neutral pictures. All stimuli were displayed for 500 ms and 1000 ms after a 2-second black screen interval, followed by a 2-second fixation cross (eFigure 2 and eMethods 3 in Supplement 2).

### Dot-Probe Task

For the dot-probe task, we used 60 picture pairs: 20 NSSI-related paired with neutral (NSSI condition), 20 trauma-related paired with neutral (trauma condition), and 20 combining NSSI and trauma-related (NSSI plus trauma condition). Each pair was presented twice, once for 200 ms (block 1) and once for 500 ms (block 2). See eMethods 4 in Supplement 2 for a detailed paradigm description. All stimuli from the free-viewing and dot-probe tasks are available on the Open Science Framework.^40^

### Initial Fixation and Fixation Durations

In the free-viewing tasks, fixations were defined as periods of constant gaze within a neutral or NSSI word or picture, lasting at least 100 ms. Initial fixations referred to the first stimulus fixated upon trial onset. The percentage of initial fixations to NSSI stimuli was calculated for each participant by dividing the number of initial fixations on NSSI stimuli by the total number of trials. A higher percentage suggested an early attentional bias toward NSSI stimuli.

### Electrophysiological Measures

Heart rate was measured using a finger clip sensor on the nondominant hand’s index finger, recording blood volume pulse at 256 Hz. Skin conductance was recorded with 2 silver/silver chloride electrodes on the index and middle fingers of the same hand, sampled at 32 Hz. Both sensors were connected to a Nexus MK-II device (Nexus Sensors, MindMedia) linked to a notebook with BioTrace+ software version 2009a (Mind Media). The Nexus device was synchronized with the Tobii TX Display via a light sensor. Skin conductance data were exported and analyzed with Ledalab^41^ version 3.4.941 for Matlab (MathWorks). For preprocessing details, see eMethods 5 in Supplement 2.

### Statistical Analysis

All analyses were conducted using R version 4.1.3 (R Project for Statistical Computing),^42^ using the packages lme4^43^ and ggplot2^44^ for analyses and plotting, respectively. *T* tests were conducted to compare baseline and mental health variables (eg, age and PTSD symptoms) between groups. Mixed-effects analyses of variance (ANOVA) were conducted to analyze physiological, subjective, eye-tracking, and dot-probe outcomes across the experiment (eMethods 6 in Supplement 2). We considered 2-sided *P* values less than .05 as significant, corresponding to an α level of 5%. Data were analyzed from December 2023 to January 2024.

## Results

The final sample consisted of 50 participants, 25 of whom engaged in NSSI (mean [SD] age, 15.86 [1.14] years; 19 female participants [76%]) and 25 of whom did not (mean [SD] age, 16.40 [1.71]; 19 female participants [76%]), all identified as White. On average, NSSI participants engaged in NSSI once in the last week, 5 times in the last month, and 71 times in the last year. The most common NSSI methods were cutting (25 participants [100%]), hitting objects (13 participants [52%]), burning (12 participants [48%]), scratching (11 participants [44%]), biting (10 participants [40%]), and inserting objects under the skin (4 participants [16%]).

Scores on the Child and Adolescent Trauma Screen 2 were significantly higher in the NSSI group (mean [SD], 21.12 [9.33]) than in the control group (mean [SD], 5.64 [7.24]) (*t*_48_ = 6.55; *P* < .001). Twelve NSSI participants screened positive for possible PTSD and were assessed with the Clinician-Administered PTSD Scale for DSM-5-Child/Adolescent Version interview; 9 met PTSD criteria (*DSM-5*). The NSSI group reported higher perceived stress (*t*_48_ = 5.42; *P* < .001) and more depressive symptoms (*t*_48_ = 7.62; *P* < .001) than the control group. In the NSSI group, 15 [60%] had a preexisting mood disorder, 10 [40%] had personality disorders, and 15 [60%] were on psychiatric medication (Table).

**Table.** Participant Characteristics

Characteristic	Mean (SD)	
	NSSI	Control
Age, y	15.86 (1.14)	16.40 (1.71)
NSSI 1-week prevalence (STIBI-R)	1.16 (1.82)	0
NSSI 4-week prevalence (STIBI-R)	5.28 (7.10)	0
NSSI 1-y prevalence (STIBI-R)	71.36 (61.21)	0
Child and Adolescent Trauma Screen-2	21.12 (9.33)	5.64 (7.24)
CAPS-CA-5^a^	35.00 (12.91)	NA
Perceived Stress Scale-10	27.00 (6.67)	16.36 (7.20)
Beck Depression Inventory II	32.32 (13.45)	8.52 (7.94)
Gender, No. (%)^b^		
Female	19 (76)	19 (76)
Male	1 (4)	6 (24)
Diverse	5 (20)	0
Previous *ICD-10* diagnoses, No. (%)		
F3x Mood [affective] disorders	15 (60)	0
F4x Neurotic, stress-related and somatoform disorders	9 (36)	0
F5x Behavioral syndromes associated with physiological disturbances and physical factors	2 (8)	0
F6x Disorders of personality and behavior	10 (40)	0
F8x Pervasive and specific developmental disorders	1 (4)	0
F9x Behavioral and emotional disorders	5 (20)	0
Current psychiatric medication, No. (%)	15 (60)	0

Abbreviations: CAPS-CA-5, Clinician-Administered PTSD Scale for *Diagnostic and Statistical Manual of Mental Disorders* (Fifth Edition) Child/Adolescent Version; *ICD-10*, *International Statistical Classification of Diseases and Related Health Problems*, Tenth Revision; NA, not applicable; NSSI, nonsuicidal self-injury.

^a^ CAPS was conducted with 12 participants in the NSSI group only. Of those 12, 9 qualified for PTSD according to CAPS-CA-5.

^b^ Gender is based on participants’ self-classification.

### Free-Viewing Task: Words

**Figure 1.**
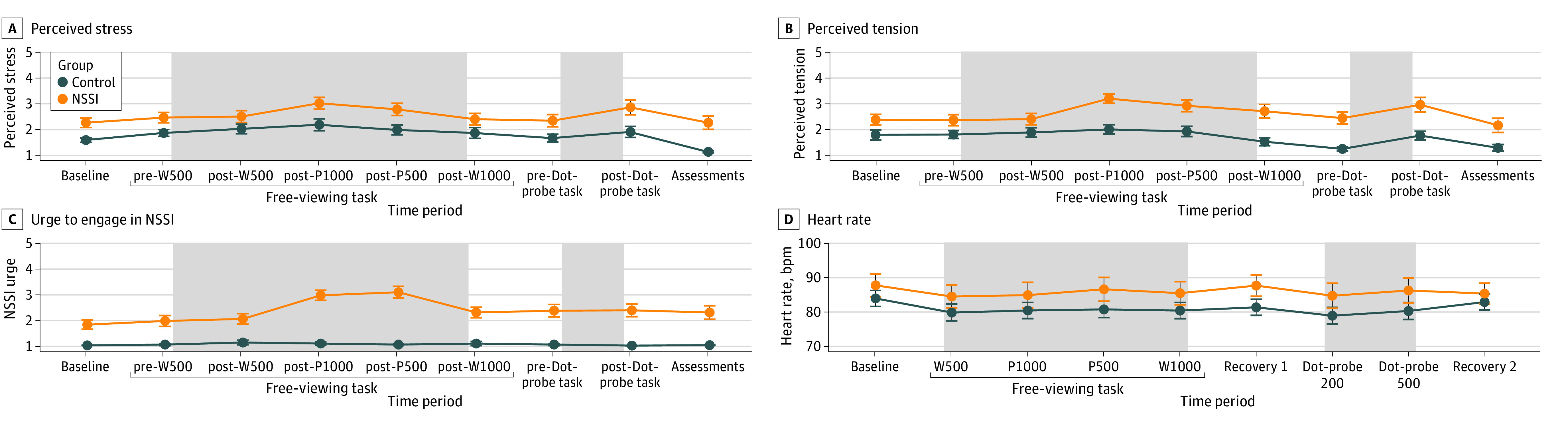
Trajectories of Perceived Stress, Perceived Tension, Urge to Engage in NSSI, and Heart Rate Over the Course of the Study A, Perceived stress: main effects of group (*P* < .001) and time (*P* < .001) significant, interaction group × time (*P* = .84) not significant. B, Perceived tension: group (*P* < .001), time (*P* < .001), group × time (*P* = .39). C, NSSI urge: group (*P* < .001), time (*P* < .001), group × time (*P* < .001). D, Heart rate: group (*P* < .001), time (*P* = .94), group × time (*P* = .10).

In the word tasks, the NSSI group reported higher stress, tension, NSSI urges, and heart rate than the control group. However, none of these values differed from baseline during the free-viewing word tasks, suggesting no elicited stress in either group (see Figure 1).

**Figure 2.**
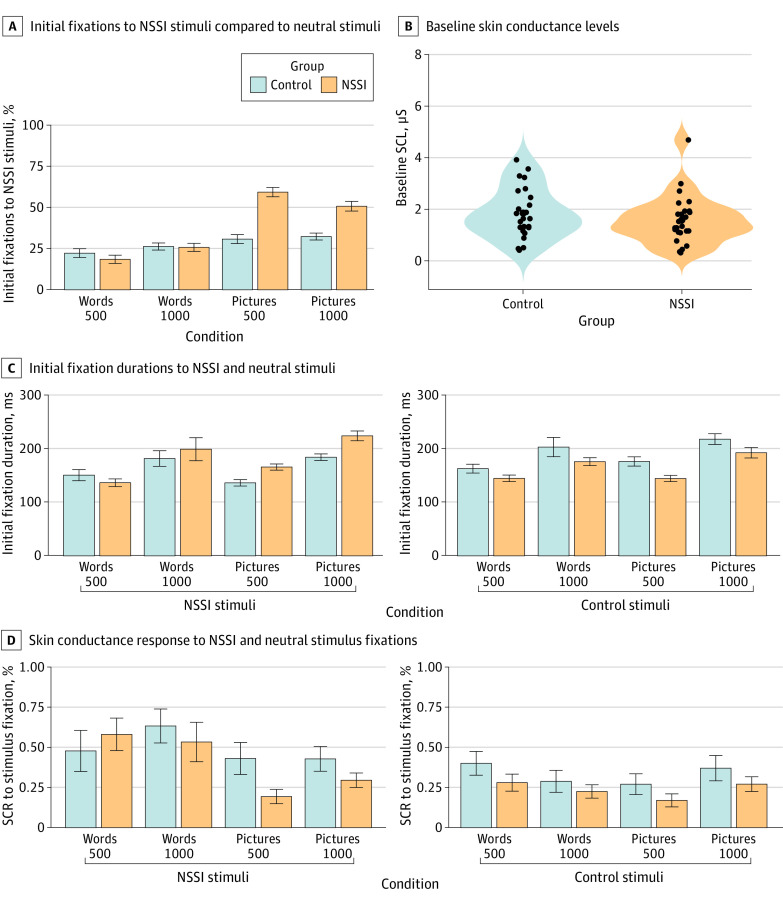
Free-Viewing Task Data A, Percentages of initial fixations on NSSI-related words and pictures. B, Baseline skin conductance levels (ie, mean 5 minutes before the task). C, Initial fixation durations on NSSI-related or neutral words and pictures. D, Percentages of skin conductance responses to NSSI-related or neutral words and pictures.

Figure 2 depicts initial fixations, fixation durations, and skin conductance responses to word stimuli. Mixed-effects ANOVAs showed no significant differences in initial fixations between individuals with NSSI and controls for NSSI-related words at 500 ms or 1000 ms. In 20% to 25% of cases (500 ms: 19% of NSSI group and 22% of control group; 1000 ms: 26% of NSSI group and 26% of control group), NSSI-related words were fixated first, suggesting random fixations. Fixation durations on NSSI-related words did not differ significantly between groups (500 ms: *P* = .80; 1000ms: *P* = .17) (Figure 2C). Skin conductance responses to initial fixations on NSSI-related words showed no significant group differences (500 ms: *P* = .19; 1000ms: *P* = .83) (Figure 2D). Thirty-seven participants (18 in NSSI group; 19 in control group) were excluded due to nonresponse, consistent with other studies.^45^ There were no baseline skin conductance differences before the task (*t* test: *P* = .33, Figure 2B).

### Free-Viewing Task: Pictures

In the free-viewing picture tasks, subjective stress, tension, and heart rate were higher in the NSSI group than in the control group, but none differed from baseline. However, a significant interaction (*F* = 3.50; *P* < .001) indicated a higher NSSI urge in the NSSI group after the 1000 ms and 500 ms tasks (Figure 1C). Post hoc tests showed this urge was significantly higher compared with the NSSI group’s baseline (*d* = 1.22; 95% CI, 0.69-1.73; *P* < .001) and the control group’s values after the 1000 ms (*d* = 2.62; 95% CI, 1.85-3.37; *P* < .001) and 500 ms tasks (*d* = 2.47; 95% CI, 1.72-3.20; *P* < .001).

In the picture tasks, the NSSI group initially fixated on NSSI-related pictures significantly more often than the control group in both 500 ms (mean difference, 28.64%; 95% CI, 18.31%-38.98%; *P* < .001) and 1000 ms conditions (18.50%; 95% CI, 9.05%-27.95%; *P* < .001), exceeding 50% of trials (Figure 2A). The NSSI group also fixated on these pictures for longer durations—29.21 ms longer in the 500 ms condition (95% CI, 4.31-54.72 ms; *P* < .001) and 39.83 ms longer in the 1000 ms condition (95% CI, 6.90-72.76 ms; *P* < .001). However, there was no difference between groups in skin conductance responses associated with initial fixations, similar to the word tasks. Seventeen participants (5 in the NSSI group and 12 in the control group) were excluded due to nonresponse.

### Attentional Bias: Dot-Probe Task

No significant increases in stress, tension, NSSI urge, or heart rate were observed during the dot-probe task (Figure 1). Response latencies deviating by more than 2 SDs and incorrect trials were excluded, removing 11.34% of trials (3.40% outliers and 78.94% incorrect).

**Figure 3.**
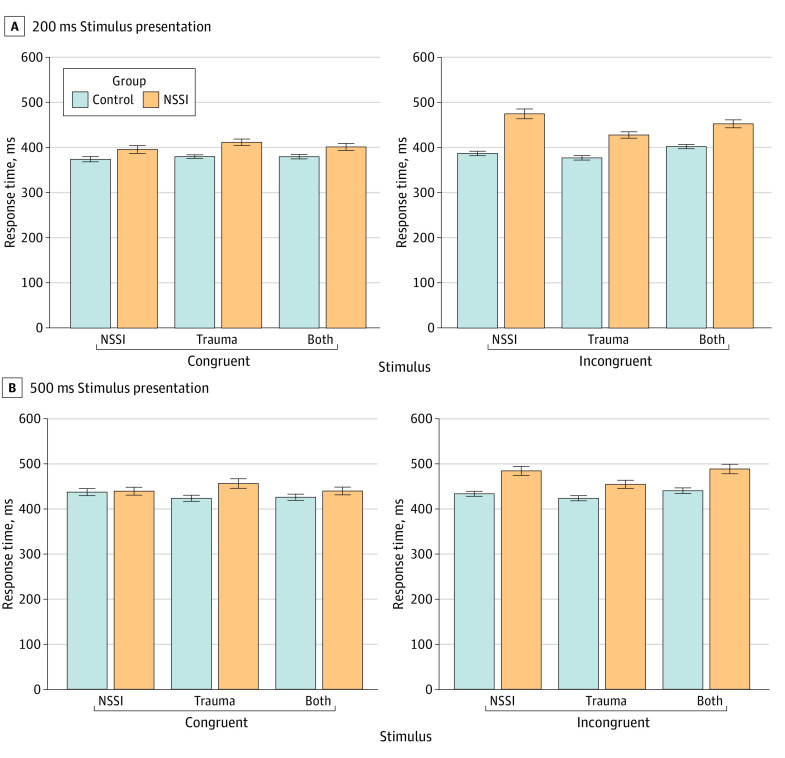
Response Times to Congruent and Incongruent Stimuli in the Dot-Probe Task A, 200 ms stimulus presentation and B, 500 ms stimulus presentation.

Mixed-effects ANOVA for 200 ms response latencies showed a significant 3-way interaction (group × stimulus × probe, *F* = 5.36; *P* = .01) (Figure 3A). Tukey post hoc tests revealed that NSSI participants responded faster to congruent than incongruent tasks. In controls, no significant differences were found. NSSI participants had longer response latencies for NSSI stimuli in congruent vs incongruent trials (mean difference, 7.94 ms; 95% CI, 46.87 to 111.02 ms; *P* < .001), and for trauma and NSSI stimuli (mean difference, 51.20 ms; 95% CI, 18.94 to 83.46 ms; *P* < .001), but not for trauma stimuli alone (mean difference, 16.30 ms; 95% CI, −14.90 to 47.50 ms; *P* = .87). Thus, the NSSI group showed an attentional bias toward NSSI stimuli, unlike the control group.

For the 500 ms stimulus presentation block, mixed-ANOVA showed a significant group × stimulus × probe interaction (*F* = 3.987; *P* = .02) (Figure 3B). NSSI participants—but not controls—had slower response times to incongruent stimuli, particularly for NSSI (mean difference, 44.98 ms; 95% CI, 6.24 to 83.72 ms; *P* = .001) and trauma and NSSI stimuli (mean difference, 48.73 ms; 95% CI, 10.53 to 86.93 ms; *P* = .002), but not for trauma stimuli (mean difference, 16.30 ms; 95% CI, −39.71 to 11.59 ms; *P* = .10). Controls showed no differences between stimuli. Thus, NSSI participants exhibited an attentional bias toward NSSI-related stimuli at 500 ms.

## Discussion

In the current study, we were able to show preliminary evidence that adolescents with a history of NSSI exhibit an attentional bias toward NSSI pictorial stimuli—though not toward NSSI textual content—and that exposure to images associated with NSSI intensifies the urge to engage in NSSI. Our study reveals that adolescents with a history of NSSI had more initial fixations and longer fixation durations on NSSI-related images than those with no history of NSSI indicating no avoidance of NSSI content. This attentional bias persisted regardless of stimulus onset duration. Additionally, the dot-probe task confirmed this bias as specific to NSSI-related stimuli, showing difficulties in disengaging from NSSI stimuli, consistent with higher fixation durations in the free-viewing task.

Images depicting wounds, scars, and emotionally charged texts are prolific on social media,^15^ leading to concerns about their potential to incite self-injurious behaviors in youth with a history of NSSI or even in those without such a history. Our study suggests an attentional bias toward NSSI-related content and subsequent increases in NSSI urges, aligning with Riquino et al,^26^ who employed a dot-probe paradigm among young adults with NSSI. Consequently, these laboratory-based insights appear to align with predominantly qualitative or retrospective findings surrounding social media usage,^17^ suggesting that engagement with image-based NSSI content on social media platforms may indeed heighten the propensity to engage in NSSI. However, adolescents with no history of NSSI in our sample did not exhibit increased urges when exposed to the same NSSI stimuli, suggesting that visual exposure alone does not necessarily trigger NSSI urges in those without a prior history of NSSI.

Furthermore, we demonstrated that NSSI-related textual stimuli did not lead to differential cognitive processing compared with neutral textual stimuli, nor did they provoke urges to engage in NSSI, regardless of a history of NSSI. Notably, cognitive research focusing on PTSD^45^ has identified cognitive biases (and physiological responses) triggered solely by trauma-related texts—a phenomenon we did not observe in our study of individuals engaging in NSSI. While further research is imperative, our findings suggest a minimal to nonexistent negative outcome of NSSI-related text.

Contrary to our hypothesis, exposure to NSSI imagery did not elevate physiological parameters such as skin conductance response or heart rate in individuals with NSSI. Skin conductance responses to NSSI-related stimuli were similar to neutral stimuli, suggesting attentional biases toward NSSI do not coincide with increased sympathetic arousal. This supports the idea that dysregulation in NSSI may occur through the parasympathetic branch, modulated by brainstem networks via vagal pathways.^46,47^ A recent meta-analysis^10^ found lower resting parasympathetic but comparable sympathetic activity in individuals with NSSI compared with controls. Without physiological or emotional arousal, urges triggered by NSSI depictions may not lead to NSSI behaviors. We also found no significant increase in stress and tension levels upon viewing NSSI images, although these levels were generally higher in participants with NSSI. Future research should explore how interpersonal stressors or other NSSI precursors affect attentional biases and physiological reactivity.

### Limitations and Strengths

Our study is not without limitations. First, our sample size is relatively small, and multiple comparisons in statistical analyses were not accounted for. Therefore, our results should be regarded as preliminary and require replication. Additionally, the NSSI group consisted mainly of patients in treatment, which may limit the generalizability to nontreatment populations. This study focuses only on adolescents, so conclusions cannot be generalized to younger individuals or adults. Factors like medication use and comorbid mental disorders may have influenced our results, particularly regarding attentional processes. However, given that NSSI is a transdiagnostic entity, such factors are both prevalent and common. Future research should control for and stratify these clinical features to better represent the diversity within adolescents and the broader NSSI population. However, our study is strengthened by the inclusion of a comparison group of adolescents with no history of NSSI, a first in this area of research, to our knowledge.

## Conclusions

In conclusion, our study suggests that adolescents who engage in NSSI exhibit an attentional bias toward NSSI-related pictures, with more initial fixations, longer durations, and difficulty disengaging. This bias was not observed with words or trauma-related images and was associated with increased NSSI urges. Importantly, attentional bias was associated with an increased urge to engage in NSSI. Given the role of NSSI depictions in potentially increasing urges, interventions that bolster emotion regulation skills, such as Dialectical Behavior Therapy—evidence-based in this clinical area—are crucial.^48,49^ For clinicians, it is essential to understand the potential for NSSI depictions to elicit urges. We encourage professionals working with individuals with a history of NSSI not only to inquire about their consumption of social media NSSI imagery but also to enhance stress management strategies as well as social media skills.

## Notes

Supplement 1.
Trial Protocol

Supplement 2. **eMethods 1. **Recruitment, Inclusion Criteria, Participant Flow, and Power Analysis **eMethods 2.** Measures **eMethods 3.** Free-Viewing Task Paradigm **eMethods 4.** Dot-Probe Task Paradigm **eMethods 5.** Preprocessing of Skin Conductance Level Responses **eMethods 6.** Analysis Strategy **eFigure 1.** TREND Flow Chart of Enrollment Details **eFigure 2.** Overview of Study Procedure and Timeline and Overview of the 2 Free-Viewing Tasks and the Dot-Probe Task
eReferences

Supplement 3.
Data Sharing Statement

## References

[1] Lloyd-Richardson EE, Perrine N, Dierker L, Kelley ML. Characteristics and functions of non-suicidal self-injury in a community sample of adolescents. Psychol Med. 2007;37(8):1183-1192. doi:10.1017/S003329170700027X 17349105 PMC2538378

[2] Plener PL, Kaess M, Schmahl C, Pollak S, Fegert JM, Brown RC. Nonsuicidal self-injury in adolescents. Dtsch Arztebl Int. 2018;115(3):23-30. doi:10.3238/arztebl.2018.0023 29366448 PMC5787659

[3] American Psychiatric Association. Diagnostic and Statistical Manual of Mental Disorders. 5th ed. American Psychiatric Association; 2013.

[4] Farkas BF, Takacs ZK, Kollárovics N, Balázs J. The prevalence of self-injury in adolescence: a systematic review and meta-analysis. Eur Child Adolesc Psychiatry. 2023. doi:10.1007/s00787-023-02264-y 37486387 PMC11564408

[5] Swannell SV, Martin GE, Page A, Hasking P, St John NJ. Prevalence of nonsuicidal self-injury in nonclinical samples: systematic review, meta-analysis and meta-regression. Suicide Life Threat Behav. 2014;44(3):273-303. doi:10.1111/sltb.12070 24422986

[6] Whitlock J, Eckenrode J, Silverman D. Self-injurious behaviors in a college population. Pediatrics. 2006;117(6):1939-1948. doi:10.1542/peds.2005-2543 16740834

[7] Castellví P, Lucas-Romero E, Miranda-Mendizábal A, . Longitudinal association between self-injurious thoughts and behaviors and suicidal behavior in adolescents and young adults: a systematic review with meta-analysis. J Affect Disord. 2017;215:37-48. doi:10.1016/j.jad.2017.03.035 28315579

[8] Kleindienst N, Bohus M, Ludäscher P, . Motives for nonsuicidal self-injury among women with borderline personality disorder. J Nerv Ment Dis. 2008;196(3):230-236. doi:10.1097/NMD.0b013e3181663026 18340259

[9] Taylor PJ, Jomar K, Dhingra K, Forrester R, Shahmalak U, Dickson JM. A meta-analysis of the prevalence of different functions of non-suicidal self-injury. J Affect Disord. 2018;227:759-769. doi:10.1016/j.jad.2017.11.073 29689691

[10] Goreis A, Prillinger K, Bedus C, . Physiological stress reactivity and self-harm: a meta-analysis. Psychoneuroendocrinology. 2023;158:106406. doi:10.1016/j.psyneuen.2023.106406 37783020

[11] Kaess M, Hooley JM, Klimes-Dougan B, . Advancing a temporal framework for understanding the biology of nonsuicidal self- injury: an expert review. Neurosci Biobehav Rev. 2021;130:228-239. doi:10.1016/j.neubiorev.2021.08.022 34450182 PMC8783544

[12] Valencia-Agudo F, Burcher GC, Ezpeleta L, Kramer T. Nonsuicidal self-injury in community adolescents: a systematic review of prospective predictors, mediators and moderators. J Adolesc. 2018;65(1):25-38. doi:10.1016/j.adolescence.2018.02.012 29522914

[13] Bentley KH, Nock MK, Barlow DH. The four-function model of nonsuicidal self-injury: key directions for future research. Clin Psychol Sci. 2014;2(5):638-656. doi:10.1177/2167702613514563

[14] Nock MK. Why do people hurt themselves? New insights into the nature and functions of self-injury. Curr Dir Psychol Sci. 2009;18(2):78-83. doi:10.1111/j.1467-8721.2009.01613.x 20161092 PMC2744421

[15] Brown RC, Fischer T, Goldwich AD, Keller F, Young R, Plener PL. #cutting: Non-suicidal self-injury (NSSI) on Instagram. Psychol Med. 2018;48(2):337-346. doi:10.1017/S0033291717001751 28705261

[16] Dyson MP, Hartling L, Shulhan J, . A systematic review of social media use to discuss and view deliberate self-harm acts. PLOS ONE. 2016;11(5):e0155813. doi:10.1371/journal.pone.0155813 27191728 PMC4871432

[17] Susi K, Glover-Ford F, Stewart A, Knowles Bevis R, Hawton K. Research review: viewing self-harm images on the internet and social media platforms: systematic review of the impact and associated psychological mechanisms. J Child Psychol Psychiatry. 2023;64(8):1115-1139. doi:10.1111/jcpp.13754 36940718

[18] Jacob N, Evans R, Scourfield J. The influence of online images on self-harm: a qualitative study of young people aged 16-24. J Adolesc. 2017;60:140-147. doi:10.1016/j.adolescence.2017.08.001 28881214 PMC5614108

[19] Plener PL, Bubalo N, Fladung AK, Ludolph AG, Lulé D. Prone to excitement: adolescent females with non-suicidal self-injury (NSSI) show altered cortical pattern to emotional and NSS-related material. Psychiatry Res. 2012;203(2-3):146-152. doi:10.1016/j.pscychresns.2011.12.012 22901627

[20] Seko Y, Kidd SA, Wiljer D, McKenzie KJ. On the creative edge: exploring motivations for creating non-suicidal self-injury content online. Qual Health Res. 2015;25(10):1334-1346. doi:10.1177/1049732315570134 25662942

[21] Bar-Haim Y, Lamy D, Pergamin L, Bakermans-Kranenburg MJ, van IJzendoorn MH. Threat-related attentional bias in anxious and nonanxious individuals: a meta-analytic study. Psychol Bull. 2007;133(1):1-24. doi:10.1037/0033-2909.133.1.1 17201568

[22] Gotlib IH, Krasnoperova E, Yue DN, Joormann J. Attentional biases for negative interpersonal stimuli in clinical depression. J Abnorm Psychol. 2004;113(1):121-135. doi:10.1037/0021-843X.113.1.121 14992665

[23] Hankin BL, Gibb BE, Abela JRZ, Flory K. Selective attention to affective stimuli and clinical depression among youths: role of anxiety and specificity of emotion. J Abnorm Psychol. 2010;119(3):491-501. doi:10.1037/a0019609 20677838 PMC2946390

[24] Price RB, Kuckertz JM, Siegle GJ, . Empirical recommendations for improving the stability of the dot-probe task in clinical research. Psychol Assess. 2015;27(2):365-376. doi:10.1037/pas0000036 25419646 PMC4442069

[25] Tonta KE, Howell J, Boyes M, McEvoy P, Hasking P. An experimental investigation of biased attention in non-suicidal self-injury: the effects of perfectionism and emotional valence on attentional engagement and disengagement. J Behav Ther Exp Psychiatry. 2023;81:101856. doi:10.1016/j.jbtep.2023.101856 36996628

[26] Riquino MR, Reese SE, Garland EL. Assessing attentional bias toward nonsuicidal self-injury cues in young adults with histories of engaging in self-harm. Child Adolesc Social Work J. 2021;38(6):641-650. doi:10.1007/s10560-020-00692-2

[27] Brausch AM, Clapham RB, Littlefield AK. Identifying specific emotion regulation deficits that associate with nonsuicidal self-injury and suicide ideation in adolescents. J Youth Adolesc. 2022;51(3):556-569. doi:10.1007/s10964-021-01525-w 34686951 PMC9554798

[28] Wang Y, Zhou Y, Li G, . Executive functions in non-suicidal self-injury comorbid first-episode and drug-naïve depression among adolescents. Psychiatry Res. 2023;328:115476. doi:10.1016/j.psychres.2023.115476 37708804

[29] Armstrong T, Olatunji BO. Eye tracking of attention in the affective disorders: a meta-analytic review and synthesis. Clin Psychol Rev. 2012;32(8):704-723. doi:10.1016/j.cpr.2012.09.004 23059623 PMC3556338

[30] Cisler JM, Koster EHW. Mechanisms of attentional biases towards threat in anxiety disorders: an integrative review. Clin Psychol Rev. 2010;30(2):203-216. doi:10.1016/j.cpr.2009.11.003 20005616 PMC2814889

[31] Rosen D, Price RB, Silk JS. An integrative review of the vigilance-avoidance model in pediatric anxiety disorders: are we looking in the wrong place? J Anxiety Disord. 2019;64:79-89. doi:10.1016/j.janxdis.2019.04.003 31051420

[32] Des Jarlais DC, Lyles C, Crepaz N; TREND Group. Improving the reporting quality of nonrandomized evaluations of behavioral and public health interventions: the TREND statement. Am J Public Health. 2004;94(3):361-366. doi:10.2105/AJPH.94.3.361 14998794 PMC1448256

[33] Fox KR, Harris JA, Wang SB, Millner AJ, Deming CA, Nock MK. Self-injurious thoughts and behaviors interview-revised: development, reliability, and validity. Psychol Assess. 2020;32(7):677-689. doi:10.1037/pas0000819 32324021

[34] Sachser C, Berliner L, Risch E, . The Child and Adolescent Trauma Screen 2 (CATS-2)—validation of an instrument to measure DSM-5 and ICD-11 PTSD and complex PTSD in children and adolescents. Eur J Psychotraumatol. 2022;13(2):2105580. doi:10.1080/20008066.2022.2105580 35928521 PMC9344962

[35] Pynoos R, Weathers F, Steinberg A, . Clinician-Administered PTSD Scale for DSM-5-Child/Adolescent Version (CAPS-CA-5). National Center for PTSD. Accessed June 18, 2024. https://www.ptsd.va.gov/professional/assessment/child/caps-ca.asp

[36] Klein EM, Brähler E, Dreier M, . The German version of the Perceived Stress Scale—psychometric characteristics in a representative German community sample. BMC Psychiatry. 2016;16(1):159. doi:10.1186/s12888-016-0875-9 27216151 PMC4877813

[37] Cohen S, Kamarck T, Mermelstein R. A global measure of perceived stress. J Health Soc Behav. 1983;24(4):385-396. 6668417

[38] Kühner C, Bürger C, Keller F, Hautzinger M. Reliabilität und validität des revidierten Beck- Depressionsinventars (BDI-II). Befunde aus deutschsprachigen stichproben. Nervenarzt. 2007;78(6):651-656. doi:10.1007/s00115-006-2098-7 16832698

[39] Beck AT. Manual for the Beck Depression Inventory-II. Psychological Corporation; 1996.

[40] Goreis A. Torn. Center for Open Science. Accessed June 18, 2024. https://osf.io/6pjrn/

[41] Benedek M, Kaernbach C. A continuous measure of phasic electrodermal activity. J Neurosci Methods. 2010;190(1):80-91. doi:10.1016/j.jneumeth.2010.04.028 20451556 PMC2892750

[42] R Core Team. R: a language and environment for statistical computing. Accessed June 18, 2024. http://www.r-project.org/

[43] Bates D, Mächler M, Bolker BM, Walker SC. Fitting linear mixed-effects models using lme4. J Stat Softw. 2015;67(1). doi:10.18637/jss.v067.i01

[44] Wickham H. Ggplot2: Elegant Graphics for Data Analysis. 2nd ed. Springer; 2016. doi:10.1007/978-3-319-24277-4.

[45] Felmingham KL, Rennie C, Manor B, Bryant RA. Eye tracking and physiological reactivity to threatening stimuli in posttraumatic stress disorder. J Anxiety Disord. 2011;25(5):668-673. doi:10.1016/j.janxdis.2011.02.010 21477983

[46] Crowell SE, Beauchaine TP, McCauley E, Smith CJ, Stevens AL, Sylvers P. Psychological, autonomic, and serotonergic correlates of parasuicide among adolescent girls. Dev Psychopathol. 2005;17(4):1105-1127. doi:10.1017/S0954579405050522 16613433

[47] Koenig J, Lischke A, Bardtke K, . Altered psychobiological reactivity but no impairment of emotion recognition following stress in adolescents with non-suicidal self-injury. Eur Arch Psychiatry Clin Neurosci. 2023;273(2):379-395. doi:10.1007/s00406-022-01496-4 36203100 PMC10070238

[48] Kothgassner OD, Robinson K, Goreis A, Ougrin D, Plener PL. Does treatment method matter? A meta-analysis of the past 20 years of research on therapeutic interventions for self-harm and suicidal ideation in adolescents. Borderline Personal Disord Emot Dysregul. 2020;7(1):9. doi:10.1186/s40479-020-00123-9 32426138 PMC7216729

[49] Kothgassner OD, Goreis A, Robinson K, Huscsava MM, Schmahl C, Plener PL. Efficacy of dialectical behavior therapy for adolescent self-harm and suicidal ideation: a systematic review and meta-analysis. Psychol Med. 2021;51(7):1057-1067. doi:10.1017/S0033291721001355 33875025 PMC8188531

