# Supplementary material for: Attentional Biases and Nonsuicidal Self-Injury Urges in Adolescents
Source: JAMA Netw Open. 2024 Jul 18;7(7):e2422892. doi: 10.1001/jamanetworkopen.2024.22892 (PMC11258595; doi:10.1001/jamanetworkopen.2024.22892)
Supplement: Supplement 1. — Trial Protocol [file jamanetwopen-e2422892-s001.pdf]

Supplement 1: Study Protocol TORN-Project

Project Information
**Title: Triggers Online Resulting in Non-suicidal Self-Injury**
**Acronym: TORN**
**DRKS-ID: DRKS00025905**
**Study Type: Experimental Study, Clinical**
**Registration type: Prospective**

Brief summary in lay language

Non-suicidal self-injury (NSSI) is a major mental health concern for adolescents. To date, only factors that can trigger NSSI via digital media (e.g., via social media) have been poorly studied. Therefore, 50 (25 with NSSI and 25 healthy) adolescents aged 14 to 18 years will participate in a diary study and provide information about their emotional state, their internet usage and specific events over the duration of one week. This is to identify events in their daily lives that may lead to NSSI. In a further step, the same adolescents will participate in a second study. In this study, triggers for NSSI (e.g. certain words, details in pictures) will be analyzed by measuring eye movements. A final study will include 50 participants with NSSI (including the existing 25 participants with NSSI), in which the influence of social rejection on the perception of triggers from social media channels and the associated stress will be investigated.

Brief summary in scientific language

RATIONALE: Non-suicidal self-injury (NSSI) represents a major worldwide mental health concern in adolescents. One of the underlying mechanisms of NSSI are the negative biases in processing social feedback from others. Especially the friendship groups in which peers engage in NSSI and experiences of social rejection are discussed as significant predictors for NSSI behavior. Both are prominent phenomena in digital social media, but only little is known about the triggering factors for NSSI behavior in online interactions (e.g. pictures, words, social dynamics).

AIM: We aim to address this paucity of research by conducting two laboratory studies and an ecological momentary assessment (EMA) over one week with an adolescent cohort. The three main objectives of this research project are (1) to investigate the purposes, risks, and benefits of social media related to NSSI in an everyday context using a 7-days real-life assessment, (2) to understand whether the presentation of wounds, scars, materials used for NSSI or words related to NSSI provoke arousal, behavioral activation or an urge or thoughts to NSSI, and (3) to explore if social exclusion alters the reactivity towards NSSI-related stimuli.

METHOD: The study uses EMA by means of a programmed smartphone app that allows patients to report events, affects, and experiences on a daily basis with an emphasis on social media usage and stress-related events. We will include 50 participants (n=25 patients with NSSI; n=25 healthy adolescents) in this phase of the project. Subsequently, these 50 participants will be invited to our first laboratory study using eye-tracking and psychophysiological measures during the presentation of NSSI-related stimuli to examine attentional bias, stimuli avoidance, autonomic reactivity and self-

report of perceived stress and NSSI-related cognitions. A third study will investigate the effect of social exclusion in 50 participants with a current history of NSSI on hormonal, physiological and reported subjective stress during exposure with NSSI-related material embedded in social media content.

RESULTS/INNOVATION: To our knowledge, this is the first study to investigate triggering effects of social media content in adolescents with NSSI conducting an everyday study as well as laboratory experiments. We study mechanisms that lead to NSSI using a multi-method approach including biological, behavioral and self-report measures to understand an issue that is highly relevant in clinical work.

**Health condition or problem studied**
Diagnosis:
Non-Suicidal Self-Injury
F60.3 Emotionally unstable personality disorder
Healthy volunteers:
Yes

**Interventions, Observational Groups**
Arm 1:
• Study 1: Ecological Momentary Assessment for one week with NSSI-patients.<br>• Study 2: Presenting neutral and NSSI-related stimuli for eyetracking in NSSI-patients.<br>• Study 3: Social exclusion (according to Cyberball) prior to a presentation of neutral and NSSI-related stimuli with NSSI-patients.
Arm 2:
• Study 1: Ecological Momentary Assessment for one week with healthy controls.<br>• Study 2: Presenting neutral and NSSI-related stimuli for eyetracking in healthy controls.<br>• Study 3: Social inclusion (according to Cyberball) prior to a presentation of neutral and NSSI-related stimuli with NSSI-patients.

**Endpoints**
Primary Outcome:
• Study 1: Urge/ideation to NSSI, NSSI frequency/intensity during 7 day period (EMA)<br>• Study 2: Urge/ideation to NSSI (self-rated), initial fixation & fixation time (both eye-tracking), autonomous response (SCR, HR) during experimental procedure<br>• Study 3: Cortisol stress response, heart rate response, initial fixation & fixation time (eye-tracking) during experimental procedure.
Secondary Outcome:
• Study 1: Mood, motives for NSSI (both, self-rated; ecological momentary assessment; duration: 7 days)<br>• Study 2: stress arousal/appraisal, positive and negative affect (all, self-rated), pupil dilatation (eyetracking) during experimental procedure<br>• Study 3: fundamental basic needs, stress arousal/appraisal, urge/ideation to NSSI (all, self-rated) during experimental procedure.

**Study Design**
Purpose:
**Other**

Study Type:
**Non-Interventional**
Longitudinal/cross-sectional:
Cross-sectional

Recruitment Locations

- Recruitment countries: Austria
- Number of study centers: Monocenter study
- Recruitment location(s): University Medical Center : Universitätsklinikum für Kinder- & Jugendpsychiatrie, Wien Wien
- Planned study start date: 2022-05-01
- Actual study start date: 2022-06-01
- Target Sample Size: 50 per study
- Final Sample Size: No Entry

Inclusion Criteria
Sex: All
Minimum Age: 14 Years<br>Maximum Age: 18 Years
Additional Inclusion Criteria:<br>Inclusion criteria for the clinical group are (a) good German language skills, (b) fulfilling NSSI within the past 12 month after SITBI-G, (c) aged between 14;00 and 17;11 years. Inclusion criteria for the healthy controls are (a) good German language skills and (b) aged between 14;00 and 17;11 years.

Exclusion Criteria
Exclusion criteria for the clinical group are (a) adolescents treated with cortisone or suffering from a hormonal dysfunction, (b) fulfilling criteria for an Autism Spectrum Disorder (ASD), (c) IQ < 70 based on previous IQ-tests or clinical impression, (d) any known medical illness or other severe comorbid mental disorder requiring immediate treatment, (e) acute suicidality or severe aggression. Exclusion criteria for the healthy controls are (a) evidence of any current mental disorder or physical disease, (b) adolescents treated with cortisone or suffering from a hormonal dysfunction, (c) IQ < 70 based on previous IQ-tests or clinical impression, (d) acute suicidality or severe aggression.

Addresses	
Sponsor:<br>Medizinische Universität Wien<br>Universitätsklinik für Kinder- &<br>Jugendpsychiatrie<br>Währinger Gürtel 18-20<br>1090 Wien, Austria	Principal Investigator:<br>Medizinische Universität Wien -<br>Universitätsklinik für Kinder- &<br>Jugendpsychiatrie<br>Mag. Dr. Oswald Kothgassner<br>Währinger Gürtel 18-20<br>1090 Wien, Austria
Telephone: +4314040030115<br>Contact per E-Mail: kjp@meduniwien.ac.at	Telephone: +4314040032350<br>oswald.kothgassner@meduniwien.ac.at

Sources of Monetary or Material Support
Public funding institutions financed by tax money/Government funding body (German Research Foundation (DFG), Federal Ministry of Education and Research (BMBF), etc.)
FWF Der Wissenschaftsfonds<br>Sensengasse 1<br>1090 Wien<br>Austria<br>Telephone:<br>+43-1-505 67 40

Ethics Committee
Address Ethics Committee:<br>Address:<br>Ethik-Kommission der Medizinischen Universität Wien [Ethik-Kommission der Medizinischen Universität Wien; Währinger Gürtel 18-20 1090 Wien Österreich]<br>Borschkegasse 8b/6<br>1090 Wien<br>Austria<br>Telephone:<br>004314040021470<br>http://ethikkommission.meduniwien.ac.at/
Vote of leading Ethics Committee:<br>Date of ethics committee application:<br>2019-06-04<br>Ethics committee number:<br>1651/2019<br>Vote of the Ethics Committee:<br>Approved<br>Date of the vote:<br>2019-09-19

Statistical Plan:

We will use Multilevel Modeling (MLM) for analyzing data of the EMA study (assessing differences in event- and signal-based affective responses). The data of MLM will be fitted using lm4 package (Bates et al., 2015) and lmerTest package (Kuznetsova et al., 2017). Within-person (time) change will be depicted in level 1 and a between-person submodel in level 2, dunking how affective responses will vary between groups (NSSI vs. HC). For the Experimental Study 1 task involving NSSI-stimuli, we will perform a 2 (group NSSI vs non-NSSI patients) x 2 (stimuli quality: NSSI-related vs neutral) repeated measures ANOVA on our dependent variables (accuracy and reaction times) as well as post-experimental comparisons (e.g. NSSI urge, perceived stress). Statistical analyses for Experimental Study 2 include number of initial fixations, time of fixations, salivary cortisol, HR as well as SCR and psychological questionnaire data. Differences between socially excluded and included participants are analyzed using repeated measures Analysis of Variance (rmANOVA). A priori power analysis using G*Power (Faul, Erdfelder, Lang, & Buchner, 2007) revealed that our study with a repeated measures design (main hypotheses) including n=50 participants per study will be efficiently powered to detect a medium effect size (i.e., interaction, Cohens $f = 0.25$, correlation among measurements $r = 0.50$) with a power of $1-\beta = 0.80$. Our power calculations follow the suggestions by Cummings (2014). We expect to detect large effects (=clinically relevant) in between-subject analyses. The sample size of

n=50 for each study is within the recommended range of level 2 grouping variables to establish accurate estimates in multilevel-modeling (Maas & Hox, 2005) for analyzing EMA.
