# Supplementary material for: Attentional Biases and Nonsuicidal Self-Injury Urges in Adolescents
Source: JAMA Netw Open. 2024 Jul 18;7(7):e2422892. doi: 10.1001/jamanetworkopen.2024.22892 (PMC11258595; doi:10.1001/jamanetworkopen.2024.22892)
Supplement: Supplement 2. — eMethods 1. Recruitment, Inclusion Criteria, Participant Flow, and Power Analysis eMethods 2. Measures eMethods 3. Free-Viewing Task Paradigm eMethods 4. Dot-Probe Task Paradigm eMethods 5. Preprocessing of Skin Conductance Level Responses eMethods 6. Analysis Strategy eFigure 1. TREND Flow Chart of Enrollment Details eFigure 2. Overview of Study Procedure and Timeline and Overview of the 2 Free-Viewing Tasks and the Dot-Probe Task eReferences [file jamanetwopen-e2422892-s002.pdf]

Supplemental Online Content

Goreis A, Pfeffer B, Gross CH, et al. Attentional biases and nonsuicidal self-injury urges in adolescents measured with eye-tracking and dot-probe paradigms. *JAMA Netw Open*. 2024;7(7):e2422892. doi:10.1001/jamanetworkopen.2024.22892

eMethods 1. Recruitment, Inclusion Criteria, Participant Flow, and Power Analysis

eMethods 2. Measures

eMethods 3. Free-Viewing Task Paradigm

eMethods 4. Dot-Probe Task Paradigm

eMethods 5. Preprocessing of Skin Conductance Level Responses

eMethods 6. Analysis Strategy

eFigure 1. TREND Flow Chart of Enrollment Details

eFigure 2. Overview of Study Procedure and Timeline and Overview of the 2 Free-Viewing Tasks and the Dot-Probe Task

eReferences

This supplemental material has been provided by the authors to give readers additional information about their work.

© 2024 [First author last name and initials or group name] et al. *[Journal name].*

eMethods 1. Recruitment, Inclusion Criteria, Participant Flow, and Power Analysis

The recruitment process involved various methods such as disseminating posters, utilizing social media advertisements, establishing communication with external psychiatric and psychological practitioners, and making internal announcements. To be eligible for participation in the study, individuals were required to meet certain inclusion criteria, stipulating that participants fall within the age range of 14 to 18 years. For the NSSI group, additional criteria included a history of at least five NSSI episodes during the previous year.

Furthermore, the NSSI group exclusively comprised individuals without a diagnosis of autism spectrum disorder and individuals who did not experience conditions necessitating acute treatment (e.g., acute psychosis), acute suicidality, or pronounced aggression. For the control group, participants were included if they reported no mental or physical disorders, either currently or in their past, as confirmed through interviews with participants and their caregiver(s). In both groups, a prerequisite was that participants exhibit adequate cognitive functioning, as determined by an Intelligence Quotient exceeding 70.

A total of n = 43 adolescents with NSSI and n = 58 healthy controls were screened for inclusion. In our trial registration, we aimed for a final sample size of 50 participants, i.e., 25 per group (NSSI and healthy controls), and thus stopped recruitment upon reaching that number of participants. Our sample size was determined to be similar to or greater than that of previous research utilizing similar methods and outcomes[1,2]. This sample size is also sufficiently powered to detect relevant medium effects (i.e., f = 0.25) between the two groups of participants, given a power of 80%[3].

In the NSSI group, a total of n = 43 individuals were screened, all of whom met the criteria for NSSI. Of these, n = 10 were excluded due to not responding after initial contact or losing interest (n = 8), or their legal guardian not providing written consent (n = 2). Consequently, 33 participants with NSSI were invited to participate in the study, of which n = 8 did not attend

© 2024 [First author last name and initials or group name] et al. *[Journal name].*

their scheduled appointment. No participant discontinued participation during the experiment in the NSSI group. The sample size of the final NSSI group was, therefore, $n = 25$.

In the healthy control group, a total of $n = 58$ individuals were screened. Of these, $n = 23$ were excluded for not responding after initial contact or losing interest ($n = 16$), their legal guardian not providing written consent ($n = 2$), or reporting a psychological disorder or physical illness ($n = 5$). Subsequently, 35 control participants were invited to participate in the study, of which $n = 10$ did not attend their scheduled appointment. No participant discontinued participation during the experiment in the control group. The sample size of the final control group was, therefore, $n = 25$.

© 2024 [First author last name and initials or group name] et al. *[Journal name].*

eMethods 2. Measures

Post-Traumatic Stress Disorder Symptoms

The CATS-2 evaluates PTSD symptoms based on both DSM-5 and ICD-11 criteria. It is a self-report measure suitable for use by children and adolescents aged 7 to 17. The 25 items align directly with the diagnostic criteria for PTSD as outlined in the DSM-5. The original validation study[4] determined that scores ≥21 represent a sensitive cut-off for probable PTSD. Therefore, all participants with a CATS-2 score of ≥21 underwent subsequent interviews using the German version of the Clinician-Administered PTSD Scale for DSM-5 in Children and Adolescents[5] to confirm diagnoses.

The CAPS-CA-5 is a structured interview to assess PTSD diagnoses in children aged seven and older. Similar to the widely used adult version of the CAPS-5[6], the CAPS-CA-5 assesses DSM-5 criteria for PTSD using 30 items. These items have been modified to be age-appropriate, and image-response options have been added. Ratings are made on a scale ranging from 0 (indicating the absence or no problem) to 4 (signifying a frequent occurrence or extreme problem). According to the CAPS-CA-5 symptom scoring rule, a symptom or impairment is considered present when rated with a severity of 2 or higher[7,8]. By employing the DSM-5 algorithm in conjunction with the scoring rule, it is possible to accurately determine whether the criteria for a PTSD diagnosis are met.

Perceived Stress

The German version[9] of the Perceived Stress Scale (PSS-10) by Cohen et al.[10] was used to indicate perceived stress in the last month. For example, participants were asked, 'In the last month, how often have you felt that you were unable to control the important things in your life?'. All items were rated on a 5-point scale (0 = never, 4 = always).

Depressive Symptoms

© 2024 [First author last name and initials or group name] et al. *[Journal name].*

We employed the German version[11] of the Beck Depression Inventory II[12] to evaluate depressive symptoms experienced in the preceding two weeks. The BDI-II has well-documented validity and reliability in assessing depressive symptoms in children and adolescents[e.g., 13,14]. The BDI-II comprises 21 individual questions, each offering four response options on a Likert scale, ranging from 0 to 3. A higher score on a particular item indicates a more pronounced expression of the symptom.

© 2024 [First author last name and initials or group name] et al. *[Journal name].*

eMethods 3. Free-Viewing Task Paradigm

The display durations of picture and word stimuli in the free-viewing task (i.e., 500 and 1000ms) were selected based on conventions in attentional research[15], wherein 500ms is typically employed to assess the initial orientation of attention, while 1000ms is often used to evaluate the sustained maintenance of attention. In all trials, the four stimuli (i.e., pictures or words) appeared at the top left, bottom left, top right, and bottom right quadrants of the fixation cross.

In the free-viewing word task, participants viewed the 64 stimuli twice—initially in the first presentation block with a 500ms presentation time, followed by a 1000ms presentation time (see Figure 1B in the main manuscript). In the word task, stimuli were randomized within each presentation duration block, ensuring no repetition of words occurred within the same presentation duration block. The four words presented in each trial were carefully matched based on their initial letter, word length, and frequency of occurrence. The placement of NSSI-related words (top left, bottom left, top right, bottom right) was evenly distributed across NSSI trials to prevent any potential effects due to positional preferences. As we investigated German-speaking participants, the majority of trials consisted of German words. However, to account for NSSI-related terms with greater salience in English (e.g., "cutting"), which are often encountered in social media, 25% of the trials included English words. All words were presented in capital letters, using the Calibri font size 28, and displayed in white against a black background.

In the free-viewing word task, participants also viewed the 64 stimuli twice—but first in a presentation block with a 1000ms presentation time, followed by a 500ms presentation time (see Figure 1B in the main manuscript). Picture stimuli within presentation duration blocks were randomized, ensuring no repetition of pictures in each presentation duration block, and the placement of NSSI-related pictures was evenly distributed to avoid potential biases due

© 2024 [First author last name and initials or group name] et al. *[Journal name].*

to specific positioning. Seventy-five percent of the NSSI images were self-created, depicting shards of glass, razors, and blood (utilizing artificial blood) both independently and on various body parts (arms, legs) of one of the authors (BP), who identified as a White, cisgender female. The remaining NSSI stimuli were derived from publicly available social media posts to reflect content that participants might naturally encounter, thereby ensuring ecological validity. The neutral images were all taken from the International Affective Picture System (IAPS[16]) with 25% of all picture sets displayed in grayscale. All pictures were presented on a black background.

© 2024 [First author last name and initials or group name] et al. *[Journal name].*

eMethods 4. Dot-Probe Task Paradigm

The NSSI stimuli and neutral stimuli used in our dot-probe task were the same as in the free-viewing task, while the trauma-related stimuli, selected to elicit threat-related attentional bias, consisted of images from the IAPS. Examples include a picture of a person holding a gun, a man strangling a woman, and a medical emergency scene. Twenty-five of the stimuli pairs (5 per condition) were presented in grayscale. Participants had the opportunity to take a short break between the two blocks.

Initially, a fixation cross appeared at the center of the computer display for 2 seconds and then vanished. Subsequently, pairs of stimuli were presented, always positioned on the left and right sides of the fixation cross. Following the 200ms (block 1) or 500ms (block 2) display of the two words, they disappeared, and a dot-probe replaced one of them for 100ms. Participants were instructed to press the left arrow key (if the dot appeared on the left side) or the right arrow key (if the dot appeared on the right side) on a wired keyboard (Fujitsu KB410) as swiftly and accurately as possible. The location of the keyboard was adjusted to suit the handedness of the participant. Moreover, the positioning of NSSI/trauma stimuli was counterbalanced across trials, with an equal distribution of 50% on the left and 50% on the right, while neutral stimuli appeared on the opposite side. In the NSSI + trauma condition, the placement of stimuli was similarly counterbalanced. The dot appeared after every pair of stimuli, regardless of whether they were NSSI, trauma-related, or neutral, with equal frequency. In the NSSI + trauma condition, the dot consistently followed the NSSI stimulus.

Trials in which the dot followed an NSSI/trauma stimulus (the first two conditions) were categorized as congruent trials, indicative of attention engagement with the threat, whereas trials in which the probe followed a neutral stimulus were classified as incongruent trials, representing attention disengagement from the threat. In trials featuring both NSSI and trauma stimuli simultaneously (i.e., the third condition without neutral stimuli), the probe following the

© 2024 [First author last name and initials or group name] et al. *[Journal name].*

NSSI stimuli was designated as congruent, and the probe following the trauma stimuli as incongruent. Shorter response latencies to a probe appearing in the prior location of an NSSI stimulus, compared to a neutral stimulus (conditions 1 and 2) or a trauma-related stimulus (condition 3), suggest heightened vigilance toward NSSI stimuli (conditions 1 and 2). In contrast, longer response latencies to a probe replacing a preceding neutral or trauma stimulus, relative to a preceding NSSI stimulus, indicate difficulty in disengaging from NSSI-related stimuli. Prior to commencing the dot-probe task, participants completed 10 practice trials (5 with a 500ms and 5 with a 200ms stimulus presentation duration) comprised solely of neutral stimuli.

© 2024 [First author last name and initials or group name] et al. *[Journal name].*

eMethods 5. Preprocessing of Skin Conductance Level Responses

Preprocessing was conducted via adaptive smoothing, and the number of skin conductance responses that were elicited by trials was obtained via continuous decomposition analysis. Parameters for skin conductance responses were defined based on conventional guidelines for measuring skin conductance responses in psychophysiological research[17], i.e., the skin conductance responses needed to occur in a response window 1–4s after trial onset and required a minimum amplitude of 0.05µS. Our outcome percentage of skin conductance responses to initial fixation of NSSI or neutral stimuli indicates a higher number of skin conductance responses—and, thus, higher autonomic nervous system arousal—in response to viewing NSSI or neutral stimuli.

© 2024 [First author last name and initials or group name] et al. *[Journal name].*

eMethods 6. Analysis Strategy

To analyze physiological and subjective outcomes across the experiment, mixed-effects ANOVAs with the between-subject factor ‘group’ and the within-subject factor ‘time’ were conducted, and subjective stress, NSSI urge, and heart rate were dependent variables. In the free-viewing tasks, we also conducted mixed-effects ANOVAs, with the between-subject factor ‘group’, with the within-subject factor ‘condition’ (i.e., NSSI-related or neutral stimulus) and the dependent variables being initial fixations, fixation duration, and skin conductance responses to initial fixations. For the dot-probe analyses, mixed-effects ANOVAs were performed with the between-subject factor ‘group’, the within-subject factor ‘probe’ (i.e., the dot position, congruent and incongruent) and response times as dependent variables. In all mixed-effects models, outcomes at Level 1 were nested within participants at Level 2. In cases of missing values, we did not use any sort of missing data imputation as mixed-effects models can be applied in case of missing or unequal data. Tukey’s post-hoc tests were performed whenever significant interactions were found.

© 2024 [First author last name and initials or group name] et al. *[Journal name].*

eFigure 1. TREND Flow Chart of Enrollment Details

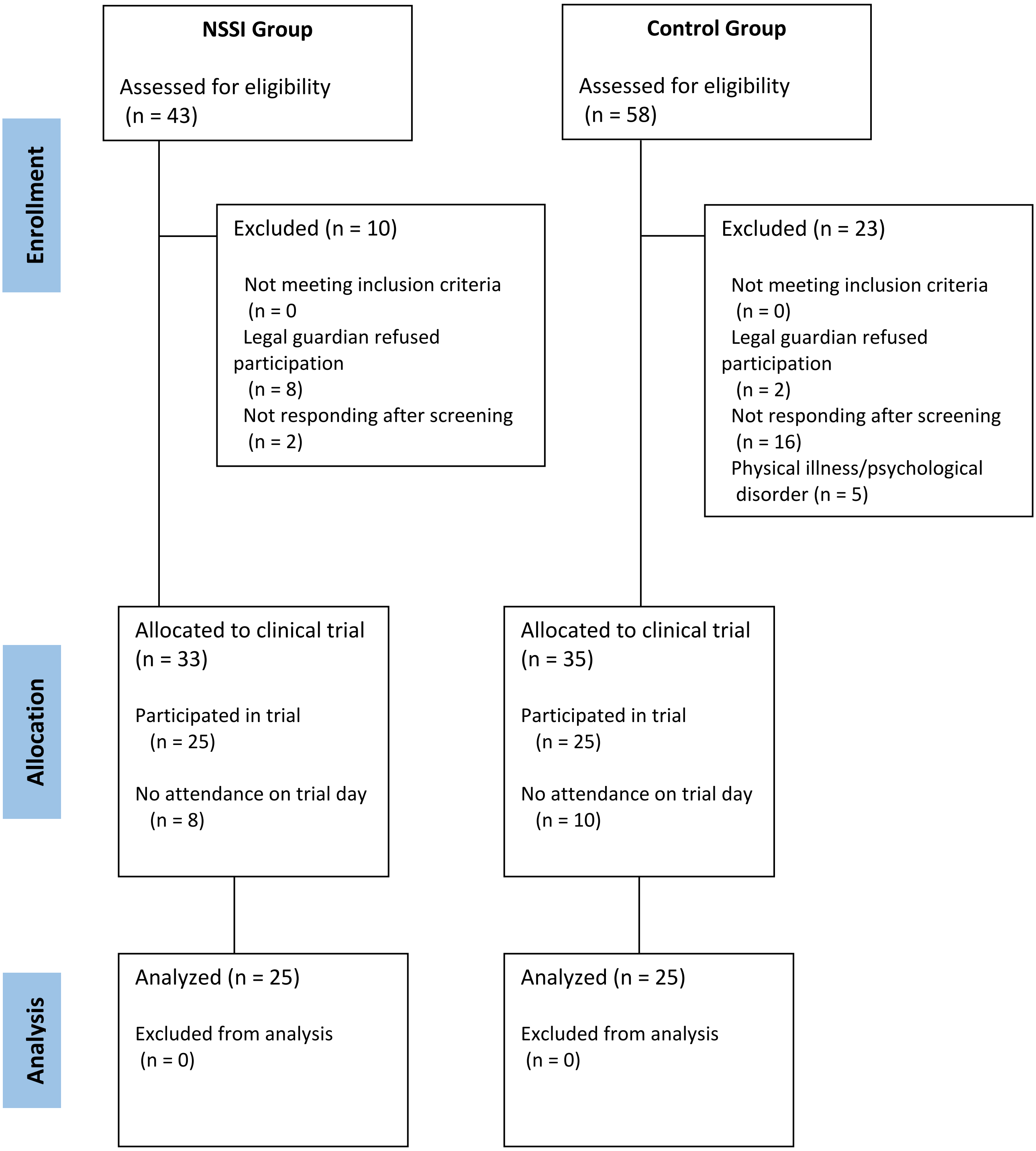

© 2024 [First author last name and initials or group name] et al. *[Journal name].*

eFigure 2. (A) Overview of Study Procedure and Timeline (B) and Overview of the 2 Free-Viewing Tasks and the Dot-Probe Task

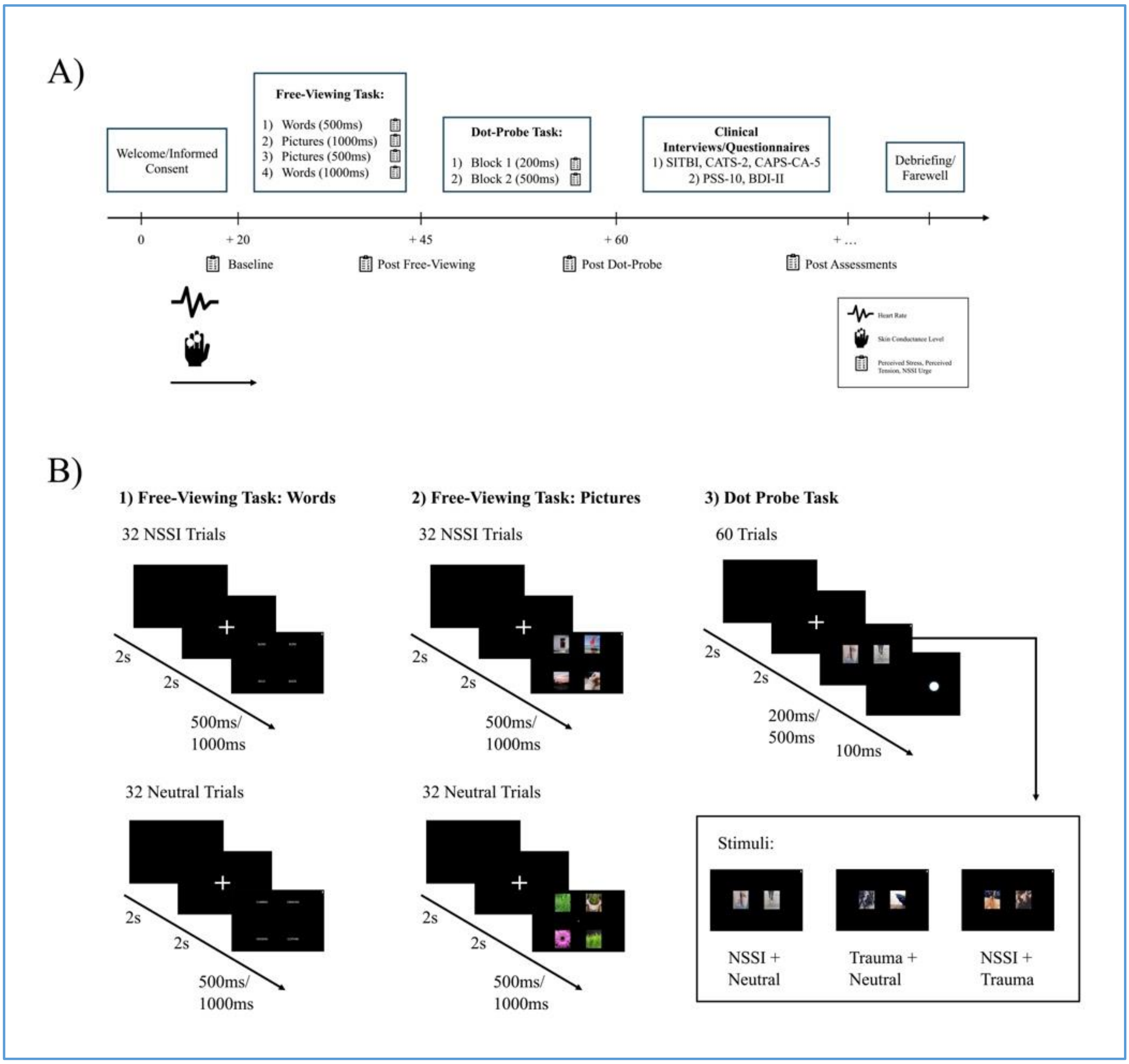

© 2024 [First author last name and initials or group name] et al. *[Journal name].*

eReferences

1. Riquino MR, Reese SE, Garland EL. Assessing Attentional Bias Toward Nonsuicidal Self-Injury Cues in Young Adults with Histories of Engaging in Self-Harm. *Child Adolesc Soc Work J*. 2021;38(6):641-650. doi:10.1007/s10560-020-00692-2
2. Felmingham KL, Rennie C, Manor B, Bryant RA. Eye tracking and physiological reactivity to threatening stimuli in posttraumatic stress disorder. *J Anxiety Disord*. 2011;25(5):668-673. doi:10.1016/j.janxdis.2011.02.010
3. Faul F, Erdfelder E, Lang AG, Buchner A. G*Power 3: A flexible statistical power analysis program for the social, behavioral, and biomedical sciences. *Behav Res Methods*. 2007;39(2):175-191. doi:10.3758/BF03193146
4. Sachser C, Berliner L, Risch E, et al. The child and Adolescent Trauma Screen 2 (CATS-2) – validation of an instrument to measure DSM-5 and ICD-11 PTSD and complex PTSD in children and adolescents. *Eur J Psychotraumatology*. 2022;13(2):2105580. doi:10.1080/20008066.2022.2105580
5. Pynoos R, Weathers F, Steinberg A, et al. Clinician-Administered PTSD Scale for DSM-5-Child/Adolescent Version (CAPS-CA-5). *Natl Cent Posttraumatic Stress Disord Natl Cent Child Trauma*. Published online 2015.
6. Weathers FW, Bovin MJ, Lee DJ, et al. The Clinician-Administered PTSD Scale for DSM–5 (CAPS-5): Development and initial psychometric evaluation in military veterans. *Psychol Assess*. 2018;30(3):383-395. doi:10.1037/pas0000486
7. Barroca I, Velosa A, Cotovio G, et al. Translation and Validation of the Clinician Administered PTSD Scale (CAPS-CA-5) for Portuguese Children and Adolescents. *Acta Médica Port*. 2022;35(9):652-662. doi:10.20344/amp.16718
8. Weathers FW, Marx BP, Friedman MJ, Schnurr PP. Posttraumatic Stress Disorder in DSM-5: New Criteria, New Measures, and Implications for Assessment. *Psychol Inj Law*. 2014;7(2):93-107. doi:10.1007/s12207-014-9191-1

© 2024 [First author last name and initials or group name] et al. *[Journal name].*

9. Klein EM, Brähler E, Dreier M, et al. The German version of the Perceived Stress Scale - psychometric characteristics in a representative German community sample. *BMC Psychiatry*. 2016;16(1):159. doi:10.1186/s12888-016-0875-9

10. Cohen S, Kamarck T, Mermelstein R. A global measure of perceived stress. Spacaban S, Oskamp S, eds. *J Health Soc Behav*. 1983;24(4):385-396.

11. Kühner C, Bürger C, Keller F, Hautzinger M. Reliabilität und validität des revidierten Beck- Depressionsinventars (BDI-II). Befunde aus deutschsprachigen stichproben. *Nervenarzt*. 2007;78(6):651-656. doi:10.1007/s00115-006-2098-7

12. Beck AT. *Manual for the Beck Depression Inventory-II.*; 1996.

13. Goreis A, Pfeffer B, Zesch HE, et al. Conspiracy beliefs and COVID-19 guideline adherence in adolescent psychiatric outpatients: the predictive role of adverse childhood experiences. *Child Adolesc Psychiatry Ment Health*. 2023;17(1):13. doi:10.1186/s13034-022-00554-y

14. Lee A, Park J. Diagnostic Test Accuracy of the Beck Depression Inventory for Detecting Major Depression in Adolescents: A Systematic Review and Meta-Analysis. *Clin Nurs Res*. 2022;31(8):1481-1490. doi:10.1177/10547738211065105

15. Field M, Cox W. Attentional bias in addictive behaviors: A review of its development, causes, and consequences. *Drug Alcohol Depend*. 2008;97(1-2):1-20. doi:10.1016/j.drugalcdep.2008.03.030

16. Lang PJ, Bradley MM, Cuthbert BN. *International Affective Picture System (IAPS): Affective Ratings of Pictures and Instruction Manual*. NIMH, Center for the Study of Emotion & Attention Gainesville, FL; 2005.

17. Boucsein W, Fowles DC, Grimnes S, et al. Publication recommendations for electrodermal measurements. *Psychophysiology*. 2012;49(8):1017-1034. doi:10.1111/j.1469-8986.2012.01384.x

© 2024 [First author last name and initials or group name] et al. *[Journal name].*
