# Supplementary material for: Attentional Biases and Nonsuicidal Self-Injury Urges in Adolescents
Source: JAMA Netw Open. 2024 Jul 18;7(7):e2422892. doi: 10.1001/jamanetworkopen.2024.22892 (PMC11258595; doi:10.1001/jamanetworkopen.2024.22892)
Supplement: Supplement 3. — Data Sharing Statement [file jamanetwopen-e2422892-s003.pdf]

Data Sharing Statement

Goreis. Attentional Biases and Nonsuicidal Self-Injury Urges in Adolescents Measured With Eye-Tracking and Dot-Probe Paradigms. *JAMA Netw Open*. Published July 18, 2024. doi:10.1001/jamanetworkopen.2024.22892

Data
Data available: Yes
Data types: Deidentified participant data
How to access data: Deidentified data from this study will be made available upon request without undue reservation.
When available: With publication

Supporting Documents
Document types: Statistical/analytic code
How to access documents: Code utilized for data analysis in this study will be shared upon request without undue reservation.
When available: With publication

Additional Information
Who can access the data: Anyone requestiing data.
Types of analyses: For any purpose.
Mechanisms of data availability: Without undue reservation.
Any additional restrictions: -
